# Time-lapse technology for embryo culture and selection

**DOI:** 10.1080/03009734.2020.1728444

**Published:** 2020-02-25

**Authors:** Kersti Lundin, Hannah Park

**Affiliations:** Reproductive Medicine, Sahlgrenska University Hospital, Göteborg, Sweden

**Keywords:** Assisted reproduction, blastocyst transfer, embryo quality, selection algorithms

## Abstract

Culturing of human embryos in optimal conditions is crucial for a successful *in vitro* fertilisation (IVF) programme. In addition, the capacity to assess and rank embryos correctly for quality will allow for transfer of the potentially ‘best’ embryo first, thereby shortening the time to pregnancy, although not improving cumulative pregnancy and live birth rates. It will also encourage and facilitate the implementation of single embryo transfers, thereby increasing safety for mother and offspring. Time-lapse technology introduces the concept of stable culture conditions, in connection with the possibility of continuous viewing and documenting of the embryo throughout development. However, so far, even when embryo quality scoring is based on large datasets, or when using the time-lapse technology, the morphokinetic scores are still mainly based on subjective and intermittent annotations of morphology and timings. Also, the construction of powerful algorithms for widespread use is hampered by large variations in culture conditions between individual IVF laboratories. New methodology, involving machine learning, where every image from the time-lapse documentation is analysed by a computer programme, looking for patterns that link to outcome, may in the future provide a more accurate and non-biased embryo selection.

## The IVF laboratory

A successful *in vitro* fertilisation (IVF) programme is to a large extent due to the laboratory conditions and the performance of the embryologist(s). A well-functioning and quality-controlled laboratory is crucial. The IVF laboratory today is a highly technical facility, with the most important features being laminar air-flow (LAF) benches, incubators, and microscopes, providing dedicated and clean areas for handling, culturing, and assessing gametes and embryos (1).

The main tasks in the lab are to optimize—as well as we currently know how—the environment and handling of gametes and embryos, and to score, rank, and select embryos to maximise the possibilities for achieving a live birth. The challenge of *in vitro* culturing of human embryos is to keep them in an environment as close to their natural environment as possible. The handling of embryos throughout the assisted reproduction technology (ART) process usually involves transferring them between dishes and assessing them at specified times (2,3). Being outside the incubator will change the culture media (pH, temperature) and thereby the embryo environment. This is believed to create a metabolic stress on the embryo (4–7) which may affect the embryo development and quality. It is therefore important that the time spent handling the oocytes and embryos outside of the controlled incubator environment is minimised. With the increasing implementation of blastocyst culture where the embryos spend a longer time period *in vitro*, a stable environment will be even more important.

Also, the static ‘snap-shot’ assessment being performed outside of the incubator once per day in traditional embryo culture means that no information is provided regarding the development between these time points, and significant events may be missed. This would mainly include abnormal cell divisions such as direct cleavage and reverse cleavage (8,9). Thereby, using only these short static assessments, some embryos would be incorrectly scored and not properly ranked for quality.

A novel technology which enables the integration of more stable embryo culture conditions with embryo assessment is the time-lapse technology. It involves the use of continuous imaging and has been introduced and implemented in ART during the last decade. This more exactly timed and electronic documentation of embryo behaviour, possibly in combination with genetic and/or metabolic analyses, is believed to contribute to embryo selection and ranking. However, the introduction of new techniques, or the change of old ones, should always be properly validated. The type of validation needed (randomised, controlled trial [RCT], observational studies, meta-analyses, in-house validation) will be dependent upon the magnitude of the change. Validation is a time-consuming and expensive process, but is crucial in order to assure safety, efficacy, and reproducibility (10,11).

## Traditional embryo culture

In the early days of IVF, a simple culture medium was used, consisting mainly of a buffered salt solution with added patient serum. This composition did not support very well the development of embryos for more than 2–3 days (12–14). In order to be able to better sustain and prolong embryo culture *in vitro*, improvement of media was essential, and the so-called sequential media were developed. The principle of these media is that the developing embryo needs to be provided with appropriate and stage-specific nutrition for each stage of development, similar to the situation as the embryo moves along the fallopian tube towards the uterus. However, with the introduction of closed culture in time-lapse units, the interest in a single medium able to support extended culture was again awakened, the principle now being that if the embryo is provided with all it needs until the blastocyst stage, it will itself utilise the correct nutrients at the correct time (15,16). Single media will thus enable an uninterrupted culture for the whole time period (17).

In a recent systematic review 20 RCTs comparing sequential and single media for blastocyst culture were included. There was no difference in ongoing or clinical pregnancy rates (18). Similar findings were reported from a meta-analysis by Dieamant et al. (19). So far it is not known which one of the two strategies is the most biologically accurate and appropriate for the embryo, and at present both types of media are in use. Clinics will base their choice of culture media on the logistics and the ways of working in the laboratory.

Thus, in the past, the common strategy was to select the ‘good-quality’ embryos (mainly looking at number of cells, degree of fragmentation, and multinucleation) at the cleavage stage, transfer 2–3 embryos, and cryopreserve the rest of the good-quality embryos, while discarding the non-good-’quality’ (judged from morphology and cleavage) embryos. Today, following the improved culture conditions in the laboratories as well as the development of more physiological culture media, extended culture until the blastocyst stage is being increasingly practised. This shift from short-term to long-term culture has been gradual, and initially many laboratories, especially in Europe, continued to perform the fresh transfer at the cleavage stage (day 2 or 3 post-fertilisation), while excess embryos were cultured to blastocyst stage and cryopreserved.

The policy of extended culture of *all* embryos, both good- and poor-quality, has led to new insights. Mainly that although ‘good-quality’ cleavage stage embryos have a higher chance of becoming good-quality blastocysts compared to ‘poor-quality’ early embryos, there is still a > 25% chance that a poor-quality embryo will become a high-quality blastocyst with the same implantation potential as a blastocyst originating from a good-quality embryo (20–22). Sadly, this implies that we have for many years discarded embryos that we now know would have had the possibility to develop to high-quality blastocysts with a good potential for implantation and live birth.

## Embryo culture in a time-lapse system

Several studies have shown that culture and handling of embryos in specialised dishes and closed time-lapse units with image capturing does not seem to impair embryo development compared to sibling oocytes cultured in a conventional setup (23–25). At the same time, there was no difference regarding fertilisation rate, embryo quality at cleavage or blastocyst stage, or ongoing pregnancy rates.

However, especially looking from laboratory logistic aspects, there are other advantages with the technology. It provides the possibility to document and assess embryo morphology and timing of developmental events through live image tracking at any time without having to move the embryo or expose it to changes in the environment. It also gives room for increased learning of ‘embryo behaviour’ (such as irregular cleavages) and the possibility to perform studies comparing for example metabolics and environment (oxygen levels, temperature, pH) to timing of specific developmental events, and in the end to success rates. The videos also enable comparisons of embryo development in different settings, such as different culture media and patient populations. In addition, time-lapse documentation may facilitate the training of embryologists in assessing embryo quality, as well as the validation of different scoring systems.

If single culture media are used, the embryos can be kept inside the time-lapse system continuously from directly after intracytoplasmic sperm injection (ICSI) or fertilisation/denudation has been performed until the day of transfer or cryopreservation. Thereby, the logistics of the IVF laboratory are simplified, and embryos can be assessed and graded at any time point during the daily workflow.

## Embryo assessment

During the evolution of embryo culture and selection strategies, a number of scoring strategies and algorithms have been developed. Most early studies were, however, based on multiple embryos for transfer, a somewhat random choice of variables and cut off levels, and/or simple univariate comparisons (26–30).

With time, as more electronic data accumulated, studies on big datasets with more advanced statistical analyses were performed. For early stage transfers, Lundin et al. showed, using stepwise logistic regression analysis of 827 day 2/3 transfers, that for ICSI embryos, early cleavage was an independent predictor of live birth (31). This was confirmed by Van Montfoort et al. who, in an analysis of 165 single embryo transfers, found that in addition to cell morphology and cell number early cleavage was an independent predictor for blastocyst development and pregnancy (32). Thurin et al. showed, in a multivariate analysis of a selected sub-analysis study group comprising 520 transfers, that 4-cell embryos resulted in a statistically higher ongoing implantation rate compared with non-4-cell embryos (33).

In 2007, Holte and his group prospectively studied 2266 IVF/ICSI double-embryo, day 2 transfers with the aim to create an evidence-based morphological embryo scoring model for prediction of implantation. The variables number of cells, variation in blastomere size, and number of mononucleated cells per embryo were found to be significant predictors for implantation. These variables were incorporated into an equation based on a 10-point integrated morphology cleavage (IMC) embryo score (34).

A follow-up prospective cohort study was performed by the same group (35). With only single embryo transfers included (*n* = 6252) and live birth rate as endpoint, a slightly revised model was constructed, where number of cells, number of mononucleated cells per embryo, and fragmentation rate were significant predictors, while variation in blastomere size was not significant. Similar findings were reached by Racowsky et al., where regression analysis of data from the American national database SART was used to standardise embryo scoring and build predictive models for day 2 and day 3 (36). Blastomere number was the most powerful predictor for implantation, while the importance of fragmentation was shown to increase for day 3 embryos compared with day 2 embryos.

As the practice of extended embryo culture increased, prediction models also for blastocysts were developed. The group of Gardner and Schoolcraft designed a blastocyst score, which became a ‘gold standard’ for blastocyst assessment. In this system, blastocysts are given a numerical score from 1 to 6 based upon their degree of expansion and hatching status. Inner cell mass (ICM) and trophectoderm (TE) are then scored, from A to C (12). However, the construction of this system is not based on multivariate statistical analyses.

In a more recent publication by Ahlström et al., the independent ability of expansion stage, inner cell mass, and trophectoderm grading to predict pregnancy outcome was studied (37). The study was a retrospective analysis of 1117 fresh day 5 single blastocyst transfers. Live birth outcome was tested to each morphological parameter. All three variables were found to have a significant effect on live birth, but, when adjusted for known confounders, only TE remained as a statistically significant independent predictor of live birth.

The same group (Ahlström et al.) (38), showed in a retrospective study of 1089 patients receiving a frozen–thawed single blastocyst transfer (*n* = 1089) that when considering pre-freeze morphology, the live birth rate increased significantly for each grade of expansion (OR 1.38, CI 1.11–1.72, *p* = 0.0041), and the probability for live birth was significantly lower for blastocysts of grade B for TE compared with grade A for TE (OR 0.68, CI 0.53–0.87, *p* = 0.0020) (38). Pre-freeze ICM morphology did not significantly predict live birth.

In addition, the chances for live birth increased for each 10% increase in degree of re-expansion after warming (*p* = 0.0042). Thus, blastocoele expansion and TE grade were selected as the most significant pre-freeze morphological predictors of live birth, and degree of re-expansion was selected as the best post-thaw parameter for prediction of live birth.

In 2013 Van den Abbeel et al. showed that in a total of 618 intracytoplasmic sperm injection patients with single-blastocyst transfer on day 5, using a simple logistic regression analysis, all three blastocyst morphology parameters were statistically significantly associated with ongoing pregnancy rates and live birth rates (*p* < 0.005 for each) (39). However, after multiple logistic regression, only blastocyst expansion stage was a significant predictor of live birth (*p* = 0.002).

## Time-lapse algorithms

The introduction of time-lapse monitoring systems, where images of embryos are captured at time intervals ranging from 5 to 20 min, has resulted in new ways of working and a modified workflow in the IVF laboratory. The technology involves different types of systems; the culture dishes and camera can be placed either as a separate system inside a regular box incubator (open system), or be completely integrated into a smaller, usually bench-top, incubator (closed system). Thus, in a closed culture time-lapse setting, the incubator will be an integrated unit with a microscope, enabling both optimised and stable culture conditions as well as direct live viewings and continuous documentation of the embryo development.

The embryos are cultured in specifically designed dishes, different for various systems, and assessed from outside the incubator via a screen. The embryos can be monitored in ‘real-time’ and viewed at the end of the culture period as a video sequence, covering the entire time of development. The different time-lapse systems have different software, and they base their scoring on differently constructed algorithms (see overview in Lundin and Ahlström) (40). It is therefore important to take into consideration that ‘time-lapse’ within ART is not a single technology, and a direct comparison of results may not always be possible.

The so-called morphokinetic variables are the morphologic and cleavage features documented at exact time points during the embryo development, such as pronuclear appearance and disappearance, cell divisions, and blastocyst formation (see more in guideline by Ciray et al.) (41). Large datasets including timing of certain development events have been analysed to create algorithms to predict implantation and live birth (42–52). Embryos have been classified using individual time-lapse morphokinetic characteristics and related to outcome measured as implantation or live birth. However, so far, the predictive power has been shown to be rather low, with AUC levels below 0.8 (43,47–52).

In an RCT by Rubio et al. (*n* = 857), a statistically increased ongoing pregnancy rate per transfer was found for the time-lapse group compared with treatments with embryos cultured traditionally (odds ratio [OR] 1.23, confidence interval [CI] 1.06–1.43) (53). However, several problems have been pointed out regarding this study, including different culture conditions for the study and interventions groups, for mixing day 5 and day 3 transfers, and for including both single and double embryo transfers. In another randomised sibling study by Yang et al. (*n* = 600), where only euploid embryos were transferred, there was a significant improvement in ongoing pregnancy rate for the embryos selected on basis of their morphokinetic scores (68.9% versus 40.5%, respectively, *p* = 0.019) (54). However, also this study had different culture conditions for the groups, making it difficult to determine if the difference observed was due to the selection method or to the culture conditions.

The most recent Cochrane meta-analysis on time-lapse technology included nine RCTs (*n* = 2955 couples) (55). The quality of the evidence ranged from very low to low. The authors conclude that there is insufficient good-quality evidence of outcome differences for embryos cultured or selected in a time-lapse system compared to traditional systems. Unfortunately, no data on cumulative live birth have been published as yet.

In addition, since embryo development variables are sensitive to environmental conditions, such as type of media, temperature, and gas levels, it has not been possible to extrapolate existing algorithms to other laboratories (56–58), and, so far, no single morphokinetic parameter has been found to be able to predict implantation potential in a multicentre setting (59,60). It is also important to consider that the much-used scoring algorithm, the KID score (known implantation data), is based on cycles where the outcome of all individual embryos is known. This means that all DET cycles with a singleton pregnancy have been excluded from the analyses that have provided the algorithm. This has to be considered a serious potential bias when embryo development is compared with outcome, especially since there is believed to exist a ‘cohort’ effect of embryos from the same patient. The question may therefore be put: had the calculated algorithms been different (and more accurate) if these non-implanting embryos had been included?

Thus, there is currently no conclusive evidence showing that selection through complex scoring systems using additive scores or constructed algorithms is more accurate on a larger scale for finding the embryo with the highest potential for implantation and live birth than the manual/visual selection by the embryologist (59).

However, even if the clinical benefit of time-lapse technology embryo culture and selection by morphokinetics is still inconclusive, another possibility is to use it as a deselection tool. For example, it has been demonstrated that certain atypical cleavage patterns, such as the occurrence of direct cleavage to three cells, negatively affects implantation (8,61). These events would in most cases be missed using traditional culture without time-lapse documentation.

It is important to note though, that despite the possibility of continuous documentation during the whole culture period, and the development of algorithms to aid in the selection, assessment and selection of embryos is still a manual and subjective intervention being performed by the embryologist. The problems with individual differences in scoring of embryo morphology, and also the annotations of exact times when certain events occur, still remain (62,63). The annotations of the kinetic data also take considerable time, and embryologists in different laboratories may annotate the same events differently, which will in turn influence the calculated score.

## Time-lapse and ploidy

A much-debated issue within IVF is the possibility of increasing live birth rates by screening the embryo for aneuploidy before transfer (preimplantation genetic testing [PGT]-A). Logically, transferring only euploid embryos should increase live birth rates through increased implantation rates and/or decreased miscarriage rates. However, so far this has been difficult to demonstrate in practice. A few RCTs using the modern techniques for PGT-A have been performed. The most recent and largest RCT, the STAR trial, including 661 treatment cycles, found no difference in ongoing pregnancy rate between the intervention group and the control group (64). The study showed ongoing pregnancy rates per intention to treat (ITT) of 41.8% (138/330) versus 43.5% (144/331) (*p* = 0.65) for the intervention group and the control group, respectively. Rates per embryo transfer were similarly equal between the groups: 50% (137/274) versus 46% (143/313) (*p* = 0.32). There was no difference in the miscarriage rates (*p* = 0.90). A total of 17% of the patients in the PGT-A group did not receive a transfer (compared to 5% in the control group) due to no available euploid blastocysts.

The current methods for performing PGT-A are highly invasive, involving removal of cells from the embryos, which could potentially influence the success rates. The time-lapse technology is an expensive tool, and much effort is put into improving its utility. It has been suggested that timing patterns could be indicative of the chromosomal status of the embryo and that algorithms could be developed for prediction of a euploid embryo. Several studies have indeed found selected morphokinetic parameters to be associated with ploidy status of the embryo (44,45,65). However, in a large cohort study, Rienzi et al. could not find any association between early (up to the 8-cell stage) morphokinetic values and aneuploidy (66). More recently, Desai et al. found that, after adjusting for female age, the late kinetic parameters tSB (time for start of blastulation), tEB (time for initiation of expansion of the blastocyst), and tEB-tSB (the time difference between these two events) were predictive of euploidy (67). The odds of a euploid blastocyst were 1.5 times higher with a tSB < 96.2 h. In addition, deselection of embryos with two or more dysmorphisms, such as multinucleation or irregular cleavage patterns, had a high predictive value. They found no predictive power in any of the early kinetic parameters.

The conflicting data between studies may suggest that the same applies for ploidy as for the time-lapse algorithms in general, i.e. the interpretations are sensitive to different patient and laboratory characteristics, and can presumably not currently be used on a universal scale.

## Future developments of time-lapse technology and embryo selection

The continuous documentation in time-lapse technology provides huge amounts of data, and only very little of it has been utilised for the existing algorithms. Current algorithms are based on annotations being made at specific times, mainly the same few traditional time points that have been used for the traditional manual assessments (41,67).

However, projects are currently ongoing using so-called deep machine learning, where large sets of time-lapse videos are analysed, not only looking at the specific predetermined morphokinetic variables, but utilising the complete accumulated data (68,69). The raw time-lapse video sequences are used as input to train and create a model. The model starts by making random predictions, which are compared to the known outcome. The deep learning system has no pre-existing assumptions as to which data should be used to build the model, but it analyses all data repeatedly through multiple layers, until a model is created that fits as closely to the known outcome as possible. In this way, subjective assessment by the embryologists are no longer involved.

In the study by Tran et al., where time-lapse videos from 8836 embryos were used to build and test a model, it was found that the trained model could predict foetal heart pregnancy from time-lapse videos with an AUC of 0.93 (95% CI 0.92–0.94) (69). The study included all cycles and embryos (fresh, cryopreserved, donated) handled during the study period and showed as a proof of principle that the deep learning model might be able to predict outcome. Future studies could refine the models by including other variables such as day of transfer or patient’s age. It was for example shown by Liu et al. that embryos with similar morphology but originating from women of different age show different implantation rates (70).

In another study by Khosravi et al., >12,000 time-lapse images from 877 good-quality embryos and 887 poor-quality embryos were used to train and implement a machine deep-learning approach to select the highest-quality embryos (68). The model was shown to predict blastocyst quality development with an AUC of >0.98 and to outperform the individual embryologists. A decision tree was developed, where the model assessed blastocyst quality integrated with patient age and associated with pregnancy outcome. Their analysis showed that chance of pregnancy depended on the blastocyst quality assessed by the model and patient age, varying from 13.8% (age ≥41 and poor-quality blastocyst) to 66.3% (age <37 and good-quality blastocyst).

## Embryo transfer

There is an on-going discussion about the best time for embryo transfer. It is argued, on the one hand, that the blastocyst better represents the correct developmental stage of the *in vivo* embryo when placed in the uterus, including a better synchrony between the blastocyst and the endometrium (see review by Teh et al.) (71). It is also argued that culture to the blastocyst stage allows for the selection of a more viable embryo, thereby resulting in increased implantation rates (72,73). However, on the other hand, assuming that current culture conditions may still be suboptimal, which would increasingly affect the embryo during the extended culture time, it is possible that some embryos may perish during the prolonged time *in vitro*. It is not known whether a good-quality cleavage stage embryo that survives to the blastocyst stage could have survived if transferred at the cleavage stage.

It is estimated that between 25% and 35% of embryos transferred at the cleavage stage implant, while for blastocyst stage transfer it is estimated to be up to 60% (2,3). These estimates were based on traditional morphology assessment, and morphokinetic variables from time-lapse documentation were not taken into consideration.

However, looking at cumulative results, including both fresh and frozen-thawed transfers from the same OPU, no difference has been shown between early and late transfer, although the early transfer requires a higher number of transfers in total to reach a live birth (74,75). Thus, being able to transfer at an earlier stage and still have the advantage of a short time to pregnancy might facilitate the work in the clinic.

In the studies by Tran et al. and Khosravi et al., only day 5 embryos were included, and so far it has not been shown if a similar model would be effective also for early cleavage stage embryos (68,69). Being able to better predict the implantation potential of an embryo already on day 2 or 3 might perhaps again change our choice for day of transfer.

## Conclusion

There is currently no conclusive evidence that ‘time-lapse technology’, mostly implying either just a closed culture system with continuous documentation and/or a combination of closed culture and morphokinetic algorithms, improves embryo quality, embryo selection, or success rates in IVF. In addition, the algorithms have not been shown to improve outcome. Nevertheless, time-lapse technology provides a very useable, although expensive, tool for the laboratory, with safe and stable culture conditions. In addition, it generates large amounts of data that will most probably aid in the selection of embryos. Further standardisation and more in-depth analyses of the large datasets available from the time-lapse documentation may be able to provide us with more targeted and stable algorithms in the future.
